# Impact of Estimated Left Atrial Pressure on Cardiac Resynchronization Therapy Outcome

**DOI:** 10.3390/jcm12154908

**Published:** 2023-07-26

**Authors:** Ahmed S. Beela, Claudia A. Manetti, Aurore Lyon, Frits W. Prinzen, Tammo Delhaas, Lieven Herbots, Joost Lumens

**Affiliations:** 1 Department of Biomedical Engineering, Cardiovascular Research Institute Maastricht (CARIM), Maastricht University Medical Centre (MUMC+), 6229 ER Maastricht, The Netherlands; c.manetti@maastrichtuniversity.nl (C.A.M.); a.lyon@maastrichtuniversity.nl (A.L.); tammo.delhaas@maastrichtuniversity.nl (T.D.); joost.lumens@maastrichtuniversity.nl (J.L.); 2 Department of Cardiovascular Diseases, Faculty of Medicine, Suez Canal University, Ismailia 41522, Egypt; 3 Department of Physiology, Maastricht University, 6200 MD Maastricht, The Netherlands; frits.prinzen@maastrichtuniversity.nl; 4 Department of Cardiology, Hartcentrum Hasselt, Jessa Hospital, 3500 Hasselt, Belgium; lieven.herbots@jessazh.be; 5 Biomedical Research Institute, Faculty of Medicine and Life Sciences, Hasselt University, 3500 Hasselt, Belgium

**Keywords:** left atrial pressure, diastolic dysfunction, cardiac resynchronization therapy, left atrial strain

## Abstract

Background: We investigated the impact of baseline left atrial (LA) strain data and estimated left atrial pressure (LAP) by applying the 2016 American Society of Echocardiography and the European Association of Cardiovascular Imaging (ASE/EACVI) guidelines on cardiac resynchronization therapy (CRT) outcomes. Methods: Datasets of 219 CRT patients were retrospectively analysed. All patients had full echocardiographic diastolic function assessment before CRT and were classified based on the guideline algorithm into normal LAP (nLAP = 40%), elevated LAP (eLAP = 49%) and indeterminate LAP (iLAP = 11%). All relevant baseline characteristics were analysed. CRT-induced left ventricular (LV) reverse remodeling was measured as the relative change of LV end-systolic volume (LVESV) at 12 ± 6 months after CRT compared to baseline. Patients were followed up for all-cause mortality for a mean of 4.8 years [interquartile range (IQR): 2.7–6.0 years]. Results: At follow-up, CRT resulted in more pronounced reduction of LVESV in patients with nLAP than in patients with eLAP. In univariate analysis, nLAP was associated with LV reverse remodelling (*p* < 0.001), as well as long-term survival after CRT (*p* < 0.01). However, multivariable analysis showed that only the association between nLAP and LV reverse remodelling after CRT is independent (*p* < 0.01). Adding LA strain analysis to the guideline algorithm improved the feasibility of LAP estimation without affecting the association between estimated LAP and CRT outcome. Conclusion: Normal LAP before CRT, estimated using the 2016 ASE/EACVI guideline algorithm, is associated with LV reverse remodelling and long-term survival after CRT. Albeit non-independent, it can serve as a non-invasive imaging-based predictor of effective therapy. Furthermore, the inclusion of LA reservoir strain in the guideline algorithm can enhance the feasibility of LAP estimation without affecting the association between LAP and CRT outcome.

## 1. Introduction

Evaluating left ventricular (LV) filling pressures is essential for establishing the diagnosis, guiding therapy, and determining prognosis of heart failure patients [1]. 

Although invasive measurements of LV filling pressures are the current gold standard, they are not routinely performed in patients. In 2016, the American Society of Echocardiography (ASE), together with the European Association of Cardiovascular Imaging (EACVI), introduced an echocardiography-based guideline algorithm for estimating left atrial pressure (LAP) [2]. By integrating echocardiographic indices, the algorithm grades the severity of diastolic dysfunction (DD) based on the estimated category of LAP, where grade I DD is linked with normal LAP (nLAP) and grades II and III are both associated with elevated LAP (eLAP). 

Several studies have validated the proposed algorithm with good accuracy against invasive LV filling pressure measurements in different cardiac pathologies, including left bundle branch block (LBBB) [3,4]. To account for missing echocardiographic data, the 2016 ASE/EACVI guideline algorithm has been supplemented with the LA reservoir strain. The combined guideline and LA strain algorithm have demonstrated similar accuracy to the guideline-only algorithm, and have also improved feasibility [5,6]. 

The impact of baseline estimated LAP on cardiac resynchronization therapy (CRT) outcome has been previously investigated with inconsistent results [7,8]. Recent data showed that grade I DD before CRT, corresponding with nLAP, is independently associated with a better CRT outcome [9]. However, the prognostic value of the combined LA + guideline algorithm has never been investigated in a CRT population.

In this retrospective single-center observational study, we investigated the association between estimated LAP and the outcome of heart failure patients treated with CRT. We aimed to: (1) investigate the impact of baseline estimated LAP, applying the 2016 ASE/EACVI guideline algorithm, on both CRT-induced LV reverse remodeling and long-term survival; (2) determine the additional prognostic value of the LA strain as a marker of LAP on heart failure patients treated with CRT.

## 2. Materials and Methods

### 2.1. Study Population

We retrospectively investigated 219 CRT patients from the database of Jessa hospital (Hasselt, Belgium). Patient inclusion was based on the availability of complete sets of baseline data (e.g., demographics, comorbidities, heart failure medications history, electrocardiogram (ECG), echocardiography, as well as survival data). We excluded 17 patients due to the bad quality of baseline echocardiography. All patients were on maximally tolerated guideline-directed heart failure medications for at least three months before CRT. Ischemic cardiomyopathy (ICM) was defined based on coronary angiography data or records of myocardial infarction. The study was approved by the Ethics Review Committee of Jessa hospital (study number: 2023-019).

### 2.2. CRT Implantation

All patients received CRT (76% with a defibrillator). LV pacing leads were positioned and guided by coronary venography, preferably in the lateral or posterolateral coronary venous branches. Device settings were optimized within a week after device implantation—as clinically appropriate—using surface ECG and Doppler echocardiography following the local protocol.

### 2.3. Electrocardiography

All patients had 12-lead surface ECG before CRT, which was digitally stored and analysed to identify rhythm and QRS width and morphology. LBBB was defined based on the criteria proposed by the 2013 European Society of Cardiology (ESC) guidelines on cardiac pacing and CRT [10].

### 2.4. Echocardiography

All echocardiographic examinations were performed using the commercially available Vivid S6 and E9 ultrasound systems (GE Healthcare, Horten, Norway). All images were digitally stored and analysed offline using the EchoPac software version 204 (GE medical systems, Horten, Norway). All patients had transthoracic echocardiography examination at 2 ± 3 months before (baseline) and 12 ± 6 months after CRT implantation (follow-up). At baseline and at follow-up, LV volumes and left ventricular ejection fraction (LVEF) were measured using the biplane Simpson’s method of disks. The severity of mitral regurgitation (MR) was quantified at baseline using the multiparametric approach proposed by the guidelines [11]. LA speckle tracking was performed at baseline on the apical 4-chamber view following the expert consensus recommendations [12]. LA strain indices (reservoir, conduit and pump strain) were automatically calculated using in-house developed software, where a zero-strain reference was defined at the onset of the QRS complex. The quality of tracking was visually checked and adjusted where needed. 

### 2.5. Diastolic Function Analysis

A full transthoracic echocardiography-based LV diastolic function analysis was performed on all patients at baseline. Mitral inflow pulsed wave (PW) Doppler was used to calculate peak E-wave velocity, E-wave deceleration time (E-DT), A-wave velocity and duration and E/A-ratio. Isovolumic relaxation time (IVRT) was measured as the time between aortic valve closure and mitral valve opening using continuous wave (CW) Doppler. Tissue Doppler Imaging (TDI) was applied to septal and lateral mitral valve (MV) annuli where septal and lateral e’ velocities were respectively recorded, and the average value of both was calculated. Tricuspid regurgitation peak velocity (TR-Vmax) was calculated using CW Doppler whenever feasible. LA volumes were calculated using the biplane Simpson’s method and indexed to body surface area (LAVi, left atrial volume indexed). 

### 2.6. Non-Invasive Estimation of Left Atrial Pressure (LAP)

**Figure 1:**
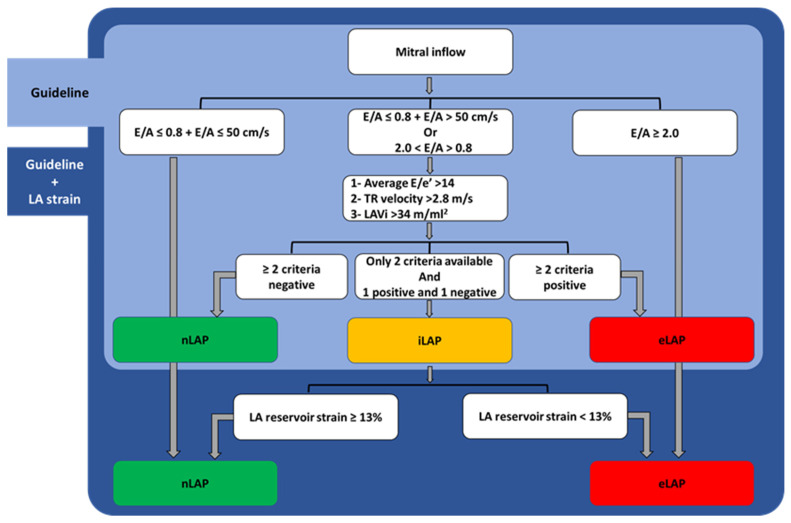
The 2016 ASE/EACVI guideline algorithm for non-invasive estimation of LAP (guideline, in the light blue box), and the guideline + LA strain algorithm (Guideline + LA strain, in the dark blue box). LAP: left atrial pressure; normal (n), indeterminate (i) and elevated (e); LAVi: left atrial volume indexed; TR: tricuspid regurgitation.

LAP was estimated at baseline using the multiparametric guideline algorithm proposed by the 2016 ASE/EACVI guideline (referred to as “guideline algorithm” in the subsequent text) [2]. In patients with more than moderate MR, as well as patients with atrial fibrillation (AF) at the time of image acquisition (total *n* = 22), additional echocardiographic parameters were used to estimate LAP, including IVRT and pulmonary venous flow analysis following the recommendations of the guidelines. Accordingly, patients were classified into three groups based on the estimated LAP category: normal (nLAP), elevated (eLAP) and, in case of missing essential echocardiographic parameter/s for categorizing LAP, an “indeterminate” (iLAP) category was assigned. We additionally used the LA reservoir strain to further determine the LAP category in the subgroup of patients with iLAP (guideline + LA strain algorithm, Figure 1) [2].

### 2.7. CRT Outcome

The relative change in LV end-systolic volume (LVESV) at follow-up compared to baseline was quantified as a continuous measure of functional response to CRT. Additionally, all patients were retrospectively followed up for all-cause mortality for a mean duration of 4.8 years [interquartile range (IQR): 2.7–6.0 years].

### 2.8. Statistical Analysis

Continuous data are expressed as mean ± standard deviation. Normally distributed data were compared between the three groups (nLAP, eLAP and iLAP) using the one-way analysis of variance (ANOVA) test for continuous variables and the Chi-square test for categorical variables. The significance of pairwise comparisons among groups was adjusted according to Bonferroni’s method. Survival rates were expressed using Kaplan–Meier’s curves, and the significance of differences in survival rates between groups was compared using a Log-rank test. The Cox proportional hazard model was used to determine predictors of survival, and the linear regression model was used to determine predictors of relative changes in LVESV at follow up. In both models, all relevant baseline variables were first tested separately in a univariate analysis and then tested all together in a multivariable model to determine variable/s with an independent association with the outcome. Additionally, to test whether the association between LAP and survival after CRT is affected by potential interaction with baseline variables, the statistical interaction between baseline nLAP and each of the variables was separately analysed in a univariate analysis. Receiver operating characteristic (ROC) with Area Under the Curve (AUC) values and Youden’s index analysis were used to identify the optimal cut-off value of the LA reservoir strain to determine the LAP category (normal vs. elevated). Data analysis was performed using SPSS (IBM Corp. Released 2015. IBM SPSS Statistics for Windows, Version 23.0. IBM Corp., Armonk, NY, USA). A two-sided *p*-value of <0.05 was considered significant.

## 3. Results

### 3.1. Baseline Characteristics of the Study Population

Applying the guideline algorithm for estimating LAP to our database, 40% of the patients had nLAP, 49% had eLAP and 11% had iLAP because of missing echocardiographic data. Supplementary Figure S1 shows he distribution of missing echocardiographic data.

The baseline characteristics of the study population and the diastolic echocardiography parameters stratified by the LAP category are summarized in Table 1. Compared to patients with eLAP, patients with nLAP were more often female, had less diabetes mellitus (DM), a lower serum creatinine and used fewer diuretics. ECG analysis showed a lower prevalence of AF and higher prevalence of LBBB morphology in patients with nLAP compared to patients with eLAP, while QRS duration was not significantly different. Patients with nLAP had a higher prevalence of mild (+1) MR, while eLAP patients showed a higher prevalence of moderately severe (+3) MR. Patients with nLAP showed better LA function on all LA strain indices (Table 1).

### 3.2. The Association between Baseline Estimated LAP and LV Reverse Remodelling after CRT

At follow-up, CRT resulted in significantly more pronounced LV reverse remodelling in patients with nLAP than in eLAP patients, as shown by a larger relative decrease in LVESV (47 ± 28 vs. 30 ± 24%, *p* < 0.01), and larger absolute increase in LVEF at follow up (17 ± 13 vs. 10 ± 19%-point, *p* = 0.02).

In univariate regression analysis, nLAP was associated with a decrease in LVESV at follow up (*p* < 0.001). In multivariable analysis, the association remained significant after adjustment of baseline variables (*p* < 0.01). LBBB was the other independent predictor of relative change of LVESV at follow up (*p* = 0.03, Table 2)**.**

Analysing LAP at CRT follow up, our data showed that 34% of patients had nLAP (*n* = 69), 33% had eLAP (*n* = 68), and 33% had iLAP (*n* = 65). Patients with nLAP both at baseline and follow up showed the most pronounced relative reduction of LVESV and absolute increase of LVEF at follow up. In contrast, patients with eLAP at both baseline and follow up showed the least pronounced relative reduction of LVESV and absolute increase of LVEF at follow up (49 ± 28 vs. 21 ± 31% decrease of LVESV, *p* < 0.001 and 21 ± 16 vs. 6 ± 20%-point LVEF, *p* < 0.01, Supplementary Figures S2 and S3). 

### 3.3. The Association between Baseline Estimated LAP and Survival after CRT 

Out of the 202 patients included in the analysis, 60 patients died during follow up. The number of deaths classified by the category of LAP is as follows: nLAP = 14, eLAP = 40 and iLAP = 6. 

**Figure 2:**
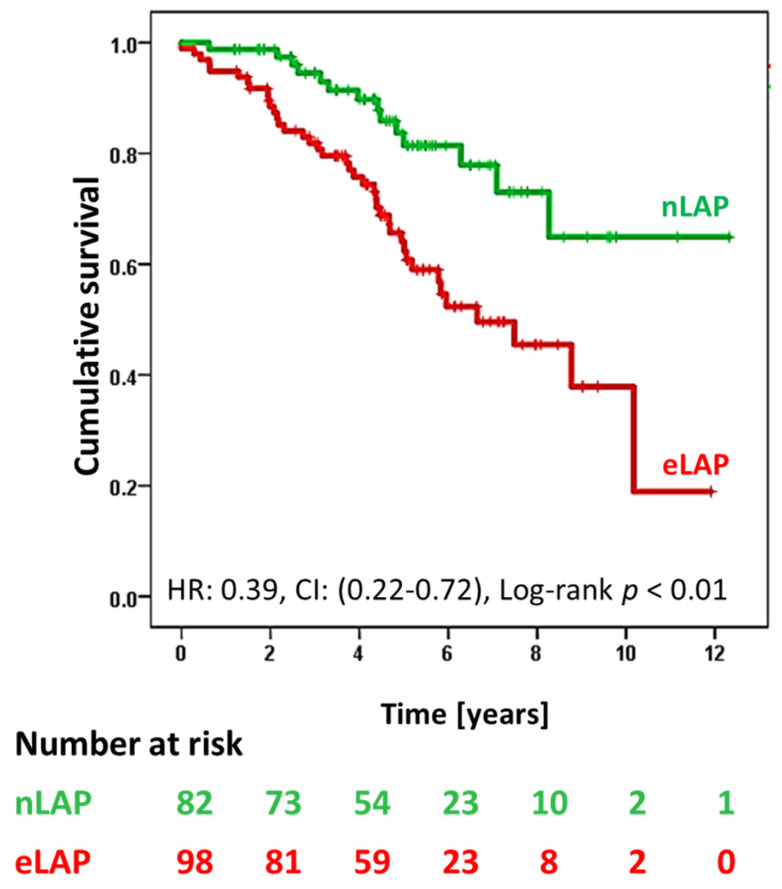
Kaplan–Meier curves depicting long-term survival of the study population after CRT implantation stratified by the estimated left atrial filling pressure (LAP) category; normal (n), indeterminate (i) and elevated (e); HR: hazard ratio; CI: confidence interval.

The survival rate after CRT was significantly higher in patients with nLAP than eLAP patients (Log-rank *p* < 0.01, Figure 2).

The univariate Cox proportional hazard analysis showed that nLAP was associated with better survival due to lower all-cause mortality [Hazard ratio (HR): 0.39, confidence interval (CI): 0.22–0.72, *p* < 0.01]. However, in multivariable analysis, the association was not significant (*p* = 0.11). Independent predictors of long-term survival were age at implantation [HR: 1.07, CI: (1.02–1.13), *p* < 0.01], AF [HR: 3.48, CI: (1.27–9.51), *p* = 0.02] and LBBB [HR: 0.42, CI: (0.20–0.86], *p* < 0.01, Table 3).

Furthermore, our data showed that there was significant statistical interaction between baseline LAP and most of the baseline variables in predicting survival after CRT, namely: age at CRT implantation, New York Heart Association (NYHA) class, serum creatinine, LBBB, QRS duration, baseline LVEF and the use of ACEi and BB (Table 4).

### 3.4. Improving the Feasibility of Estimating LAP using LA Reservoir Strain 

**Figure 3:**
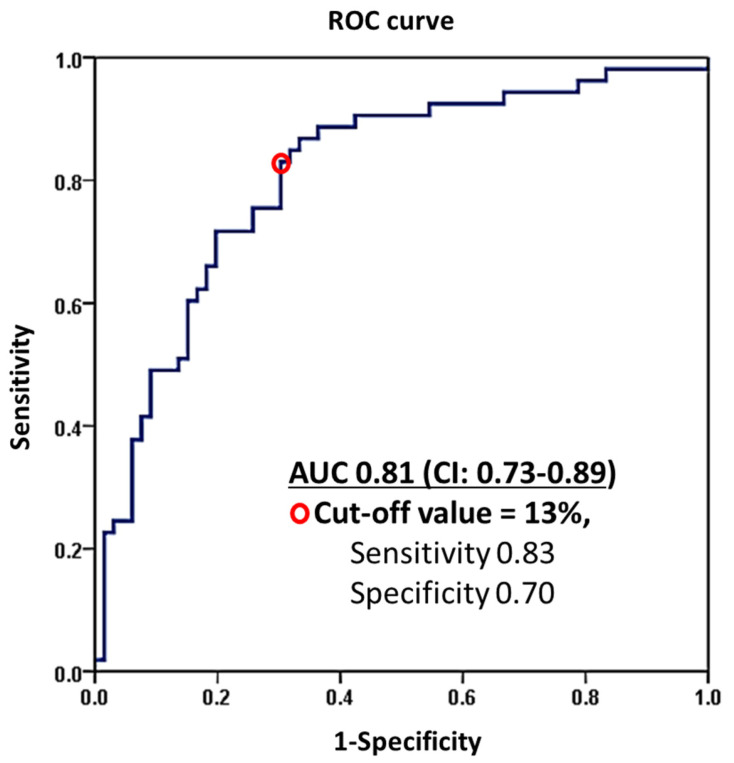
Receiver operator characteristic (ROC) curve showing the ability of LA reservoir strain to predict normal left atrial pressure (nLAP) in the entire study population. AUC: area under the curve; CI: confidence interval.

LA strain data was available in 70% of the study population (*n* = 142). Using ROC analysis, an LA reservoir strain value of 13% had a sensitivity of 0.83 and a specificity of 0.70 with an AUC of 0.81 (CI: 0.73–0.89) and Youden’s index of 0.53 for the discrimination between patients with nLAP (≥13%) and eLAP (<13%, Figure 3).

**Figure 4:**
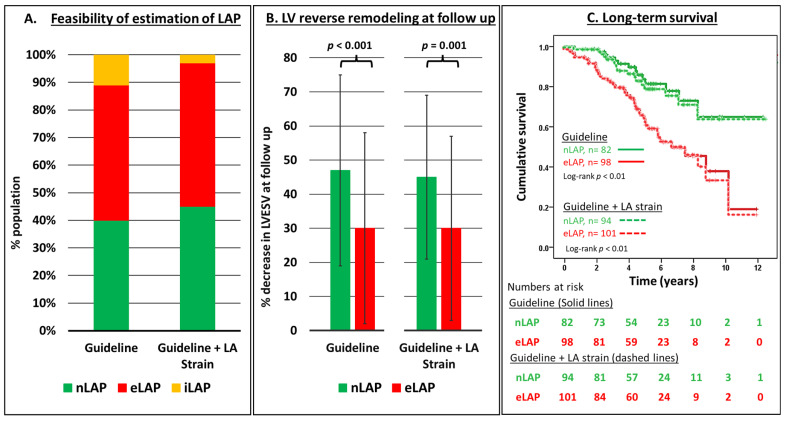
Comparing the prognostic value of both the guideline (2016 ASE/EACVI) and the guidelines + LA strain algorithms for the estimation of LAP category on (**A**) the feasibility of both algorithms, (**B**) LV reverse remodeling at CRT follow up, and (**C**) long term survival (endpoint: all-cause mortality). Note that in the guideline + LA strain algorithm, an LA reservoir strain cut-off value was used to further classify patients with iLAP into nLAP (≥13%) and eLAP (<13%). LAP: estimated left atrial pressure; normal (n), indeterminate (i) and elevated (e).

In the iLAP subgroup (*n* = 22), LA reservoir strain was accordingly used to further identify patients with nLAP and eLAP. Applying the combined guideline + LA strain algorithm decreased the size of iLAP subgroup from 11% to 3%; thus, the feasibility of estimating LAP was improved from 89% to 97% compared to the guideline algorithm (Figure 4A). Only patients with unavailable baseline LA strain data remained unclassified after applying the combined algorithm. 

Additionally, patients with nLAP and eLAP, according to the guideline + LA strain algorithm, showed the same average amount of LV reverse remodelling at follow-up (Figure 4B) and very similar survival to their counterparts according to the guideline-only algorithm (Figure 4C). Similarly, the significance of the predictive value of nLAP on both LV reverse remodelling and long-term survival after CRT in univariable and multivariable analyses was similar applying either the guideline + LA strain algorithm (Supplementary Tables S1 and S2) or the guideline algorithm only (Table 2 and Table 3). Accordingly, adding the LA strain to the guideline algorithm did not affect the association between LAP and CRT outcome.

## 4. Discussion

The main results of the current work can be summarized as follows: (1) baseline nLAP is independently associated with LV reverse remodelling at CRT follow up; (2) baseline nLAP is associated with better survival after CRT, although it was not significant in multivariable analysis; (3) supplementing the guideline algorithm with LA reservoir strain improves the feasibility of LAP estimation in a CRT population without affecting the observed association between LAP and CRT outcome.

### 4.1. The Association between Baseline Estimated LAP and LV Reverse Remodelling after CRT

We did not use a specific cut-off value of LV reverse remodelling for defining CRT response. Earlier data showed that any amount of LV reverse remodelling after CRT is associated with better survival [13]. Accordingly, we used the relative change in LVESV upon CRT as a continuous variable. 

Our data showed that baseline nLAP is independently associated with LV reverse remodelling after CRT. This finding is in line with earlier data, where baseline LAP assessed both non-invasively by echocardiography and invasively by measuring pulmonary capillary wedge pressure (PCWP) was an independent predictor of LV reverse remodelling at CRT follow up [8]. Recent data additionally showed that baseline grade I DD, which corresponds to nLAP, was associated with CRT response (defined as at least 15% reduction in LVESV after one year of CRT) [9]. 

### 4.2. The Association between Baseline Estimated LAP and Survival after CRT

Data investigating the association between baseline LAP and hard endpoints including mortality after CRT are few. Recent data showed that grade I DD (nLAP) was independently associated with a better CRT outcome [9]. However, the composition of the data in the multivariable model of that study (composite outcome of heart transplantation, LV-assisted device implantation or all-cause mortality) differed from ours, which is based on all-cause mortality only, and might yield different results. 

According to our data, the better survival of patients with nLAP compared to patients with eLAP could be explained by: (1) most favourable baseline characteristics for better CRT outcome were relatively more prevalent in nLAP than in eLAP patients including less DM, lower serum creatinine, less AF and higher prevalence of female sex and LBBB; (2) nLAP was associated with LV reverse remodelling at CRT follow up. Previous data showed that LV reverse remodeling and survival after CRT are significantly positively correlated [14,15]. Nevertheless, the lack of the significance of the association between nLAP and survival after CRT in the multivariable analysis of our study might be attributed to the relatively small sample size.

### 4.3. The Association between LA Reservoir Strain and LAP in CRT Candidates

In the work by Inoue et al., in patients with LVEF < 50%, an LA reservoir strain of <18% was able to identify patients with elevated LAP, defined as PCWP > 12 mmHg, with an accuracy of 81% [5]. However, the number of patients with LBBB in their data was too small to conclude the utility of the LA strain in evaluating LAP in patients with LBBB. In our study, 51% of the population had LBBB, which may indicate that the proposed cut-off value in their work may not be applicable to our CRT population. Additionally, following the recommendations for CRT implantation, CRT candidates have more severe heart failure, with an LVEF ≤ 35% and refractory symptoms despite medical therapy [16]. Although data investigating the association between LA reservoir strain and invasively measured LAP in a CRT population is lacking, a lower cut-off value than the previously proposed value might accordingly be expected. 

### 4.4. Limitations

The main limitations of the current work are the relatively small sample size, the retrospective design of the study, and the lack of invasive measurements of LAP. In the current work, LAP was non-invasively estimated using echocardiography not only by applying the 2016 ASE/EACVI guideline algorithm, but also using LA strain data. The combined guideline + LA strain algorithm has been previously validated; however, this was carried out with a non-CRT population [5]. Alternatively, the current work is amongst the first studies dedicated to test the prognostic value of the combined algorithm on the outcome of heart failure patients treated with CRT. In the current analysis, the association between LAP and laboratory markers of heart failure like N-terminal pro-brain natriuretic peptide (NTproBNP) was not investigated due to unavailability. In contrast, we investigated the association between baseline LAP and both functional responses to CRT in terms of LV reverse remodelling at follow up in addition to long-term survival after CRT. Although the combined algorithm improved the feasibility of LAP estimation without affecting the association between LAP and CRT outcome, a proper validation study of the combined algorithm against invasive LAP measurement in a CRT population is necessary.

## 5. Conclusions

Normal LAP before CRT, estimated using the 2016 ASE/EACVI guideline algorithm, is associated with LV reverse remodelling and long-term survival after CRT. Albeit non-independent, it can serve as a non-invasive imaging-based predictor of effective therapy. Furthermore, the inclusion of the LA reservoir strain in the guideline algorithm can enhance the feasibility of LAP estimation without affecting the association between LAP and CRT outcome. 

## Figures and Tables

**Table 1:** Baseline characteristics of the study population.

	nLAP (*n* = 82)	iLAP (*n* = 22)	eLAP (*n* = 98)	*p*-Value
**Demographics and comorbidities**				
Age at implantation (years)	69 ± 11	66 ± 10	71 ± 11	0.17
Male sex, *n*	49 (60%) ^e^	16 (72%)	77 (79%)	0.03
ICM, *n*	39 (48%)	10 (45%)	55 (56%)	0.38
NYHA class	2.7 ± 0.5	2.7 ± 0.5	2.7 ± 0.5	0.61
DM, *n*	14 (17%) ^e^	5 (23%)	29 (30%)	0.02
Serum Hb (mmol/L)	7.8 ± 1.0	7.8 ± 1.1	7.8 ± 1.2	0.21
Serum creatinine (µmol/L)	97.3 ± 53.4 ^e^	97.3 ± 26.5 ^e^	132.6 ± 53.1	<0.001
**Heart failure medications**				
ACEi, *n*	69 (84%)	17 (77%)	76 (78%)	0.25
BB, *n*	71 (86%)	19 (86%)	78 (80%)	0.20
Diuretics, *n*	47 (57%) ^e^	10 (45%) ^e^	75 (82%)	<0.01
**ECG data**				
AF, *n*	3 (4%) ^e^	0 (0%)	14 (14%)	0.01
QRS width (ms)	159 ± 18	158 ± 13	153 ± 18	0.08
LBBB, *n*	51 (62%) ^e^	12 (55%)	41 (35%)	0.04
**Echocardiographic data**				
E-velocity (m/s)	0.5 ± 0.2 ^i,e^	0.7 ± 0.2 ^e^	0.9 ± 0.3	<0.001
E-DT (msec)	265 ± 88 ^e^	230 ± 60 ^e^	160 ± 46	<0.001
A velocity (m/s)	0.8 ± 0.3 ^e^	0.9 ± 0.2 ^e^	0.6 ± 0.4	<0.001
E/A ratio	0.7 ± 0.3 ^e^	0.9 ± 0.3 ^e^	2.6 ± 4.3	<0.001
Average e’	0.5 ± 0.2	0.4 ± 0.1	0.4 ± 0.1	0.7
Average E/e’	12 ± 4 ^e^	17 ± 5	23 ± 10	<0.001
TR-VMAX (m/s)	2.3 ± 0.4 ^e^	2.2 ± 0.3 ^e^	3.0 ± 0.5	<0.001
LAVi (mL/kg/m^2^)	30 ± 15 ^e^	31 ± 12 ^e^	44 ± 18	<0.001
LVEDV (mL^3^)	166 ± 84	154 ± 62	182 ± 75	0.18
LVESV (mL^3^)	112 ± 71	99 ± 42	128 ± 65	0.09
LVEF (%)	35 ± 11	36 ± 9	32 ± 11	0.05
MR				
0, *n*	3 (4%)	2 (9%)	1 (1%)	<0.01
+1, *n*	45 (55%) ^e^	12 (55%)	42 (43%)	
+2, *n*	18 (22%)	5 (23%)	30 (31%)	
+3, *n*	1 (1%) ^e^	1 (5%)	18 (18%)	
+4, *n*	0 (0%)	1 (5%)	0 (0%)	
**LA strain data**				
LA reservoir (%)	21 ± 9 ^e^	21 ± 9 ^e^	12 ± 7	<0.001
LA conduit (%)	10 ± 4 ^e^	10 ± 6	7 ± 5	0.01
LA pump (%)	11 ± 7 ^e^	11 ± 5 ^e^	4 ± 4	<0.001

LAP: left atrial pressure; normal (n), indeterminate (i) and elevated (e); ICM: ischemic cardiomyopathy; NYHA: New York heart association; DM: diabetes mellitus; Hb: hemoglobin; ACEi: angiotensin converting enzyme inhibitors; BB: B-blockers; AF: atrial fibrillation; LBBB: left bundle branch block; E-DT: E wave deceleration time: TR-VMAX: Tricuspid regurgitation maximum velocity: LAVi: left atrial volume indexed; LVEDV: left ventricular end-diastolic volume; LVESV: left ventricular end-systolic volume; LVEF: left ventricular ejection fraction; MR: mitral regurgitation; LA: left atrial. ^e^ *p* < 0.05 vs. eLAP. ^i^ *p* < 0.05 vs. iLAP. The results are expressed as mean ± standard deviation for continuous variables and number (and percentage) for categorical variables.

**Table 2:** Univariable and multivariable linear regression analysis with relative decrease in left ventricular end-systolic volume (LVESV) as the dependent variable.

	Univariable		Multivariable	
	Standardized Coefficient	*p*-Value	Standardized Coefficient	*p*-Value
Age at implantation (years)	0.01	0.91	−0.08	0.35
Male sex	−0.19	<0.01	−0.16	0.07
ICM	−0.21	<0.01	−0.16	0.08
QRS width (msec)	0.16	0.04	0.05	0.49
NYHA	−0.12	0.09	−0.15	0.06
AF	−0.20	<0.01	−0.14	0.08
DM	−0.02	0.76	0.05	0.52
Serum Creatinine (µmol/L)	−0.11	0.15	0.12	0.18
ACEi	−0.02	0.77	−0.08	0.35
BB	0.05	0.46	0.02	0.84
LVEF (%)	−0.20	<0.01	−0.14	0.09
nLAP (vs. iLAP and eLAP)	0.26	<0.001	0.24	<0.01
LBBB	0.28	<0.001	0.17	0.03

ICM: ischemic cardiomyopathy; NYHA: New York heart association; AF: atrial fibrillation; DM: diabetes mellitus; ACEi: angiotensin converting enzyme inhibitors; BB: B-blockers; LVEF: left ventricular ejection fraction; LAP: left atrial pressure; normal (n), indeterminate (i) and elevated (e); LBBB: left bundle branch block.

**Table 3:** Univariable and multivariable Cox regression analysis for identifying predictors of survival (endpoint: all-cause mortality).

	Univariable				Multivariable	
	B	SE	Unadjusted HR (95% CI)	*p*-Value	Adjusted HR (95% CI)	*p*-Value
Age at implantation (years)	0.07	0.02	1.08 (1.04–1.11)	<0.001	1.07 (1.02–1.13)	<0.01
Male sex	0.56	0.29	1.74 (0.98–3.10)	0.06	0.99 (0.40–2.44)	0.98
ICM	0.62	0.26	1.86 (1.13–3.06)	0.02	0.78 (0.35–1.76)	0.56
QRS width (msec)	0.12	0.007	1.01 (0.99–1.03)	0.12	1.01 (0.98–1.03)	0.46
NYHA	0.49	0.24	1.63 (1.02–2.62)	0.04	1.42 (0.63–3.19)	0.40
AF	0.89	0.31	2.46 (1.33–4.54)	<0.01	3.48 (1.27–9.51)	0.02
DM	0.27	0.28	1.31 (0.76–2.27)	0.33	1.04 (0.47–2.29)	0.93
Serum Creatinine (µmol/L)	0.78	0.16	2.17 (1.58–2.98)	<0.001	1.78 (0.92–3.42)	0.08
ACEi	−0.68	0.33	0.50 (0.27–0.95)	0.04	0.23 (0.50–2.99)	0.66
BB	−0.67	0.36	0.51 (0.25–1.05)	0.07	0.79 (0.29–2.09)	0.67
LVEF (%)	−0.01	0.01	0.98 (0.97–1.12)	0.36	0.98 (0.59–1.02)	0.37
nLAP (vs. iLAP and eLAP)	−0.93	0.31	0.39 (0.22–0.72)	<0.01	0.51 (0.22–1.16)	0.11
LBBB	−0.87	0.29	0.42 (0.24–0.74)	<0.01	0.42 (0.20–0.86)	<0.01

B: Coefficient; SE: standard error; Hazard ratios (HR) are presented with 95% confidence interval (CI) in parentheses. ICM: ischemic cardiomyopathy; NYHA: New York heart association; AF: atrial fibrillation; DM: diabetes mellitus; ACEi: angiotensin converting enzyme inhibitors; BB: B-blockers; LVEF: left ventricular ejection fraction; LAP: left atrial pressure; normal (n), indeterminate (i) and elevated (e); LBBB: left bundle branch block.

**Table 4:** Univariable Cox regression analysis of the interaction between normal left atrial pressure (nLAP) and baseline variables for identifying predictors of survival (endpoint: all-cause mortality).

Interaction	*p*-Value
nLAP * Age at implantation (years)	0.01
nLAP * Male sex	0.23
nLAP * ICM	0.39
nLAP * QRS (msec)	<0.01
nLAP * NYHA	0.01
nLAP * AF	0.74
nLAP * DM	0.25
nLAP * serum Creatinine (µmol/L)	0.03
nLAP * ACEi	<0.01
nLAP * BB	<0.01
nLAP * LVEF (%)	<0.01
nLAP * LBBB	0.03

ICM: ischemic cardiomyopathy; NYHA: New York heart association; AF: atrial fibrillation; DM: diabetes mellitus; ACEi: angiotensin converting enzyme inhibitors; BB: B-blockers; LVEF: left ventricular ejection fraction; nLAP: normal left atrial pressure; LBBB: left bundle branch block.

## Data Availability

The data are available upon request from the corresponding author.

## Supplementary Materials

The following supporting information can be downloaded at: https://www.mdpi.com/article/10.3390/jcm12154908/s1. Figure S1: The distribution of the missing echocardiographic parameters in the group with indeterminate left atrial pressure (iLAP); Figure S2: The association between estimated left atrial pressure (LAP) both at baseline (BL) and follow up (FU), and CRT-induced LV reverse remodeling as expressed by the percentage decrease of left ventricular end systolic volume (LVESV) at follow up; Figure S3: The association between estimated left atrial pressure (LAP) both at baseline (BL) and follow up (FU), and CRT-induced LV reverse remodeling as expressed by the percentage point increase of left ventricular ejection fraction (LVESV) at follow up. Table S1: Univariable and multivariable linear regression analysis with relative decrease in left ventricular end-systolic volume (LVESV) as the dependent variable; Table S2: Univariable and multivariable Cox regression analysis for identifying predictors of survival (endpoint: all-cause mortality).
