# Supplementary material for: Impact of Estimated Left Atrial Pressure on Cardiac Resynchronization Therapy Outcome
Source: J Clin Med. 2023 Jul 26;12(15):4908. doi: 10.3390/jcm12154908 (PMC10419616; doi:10.3390/jcm12154908)
Supplement: Supplementary file 1 [file jcm-12-04908-s001.zip › jcm-2488753-supplementary.pdf]

Supplementary analysis

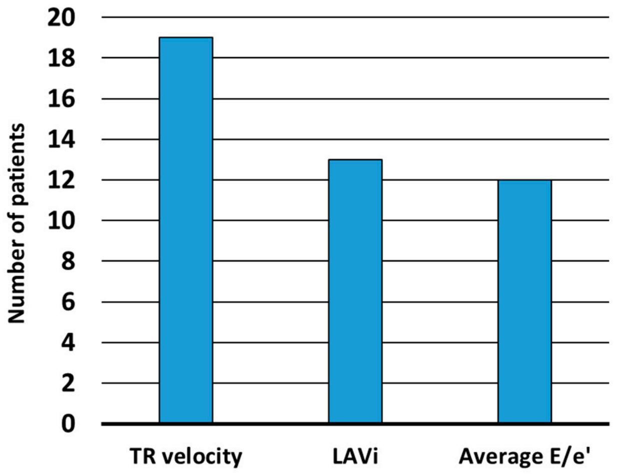

Supplementary figure S1. The distribution of the missing echocardiographic parameters in the group with indeterminate left atrial pressure (iLAP). TR: Tricuspid regurgitation, LAVi: left atrial volume indexed to body surface area.

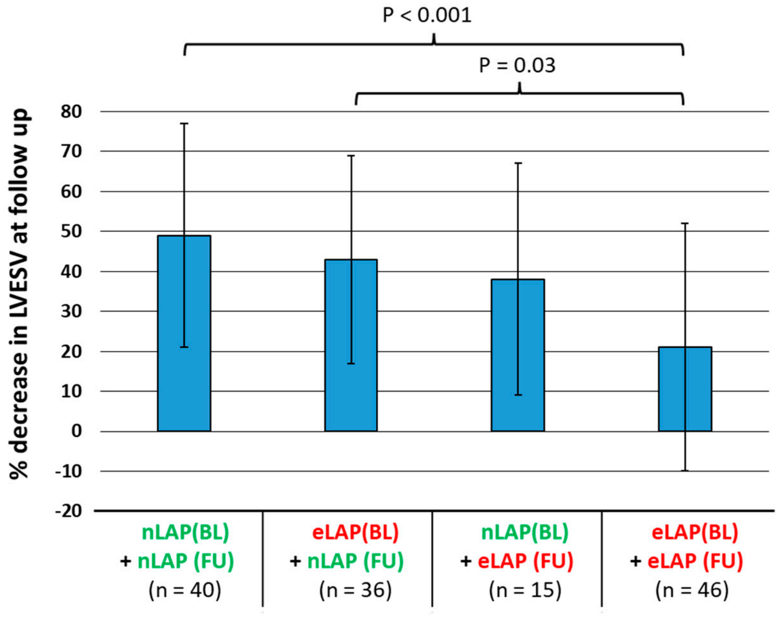

Supplementary figure S2. The association between estimated left atrial pressure (LAP) both at baseline (BL) and follow up (FU), and CRT induced LV reverse remodeling as expressed by the percentage decrease of left ventricular end systolic volume (LVESV) at follow up. eLAP: elevated LAP; nLAP: normal LAP.

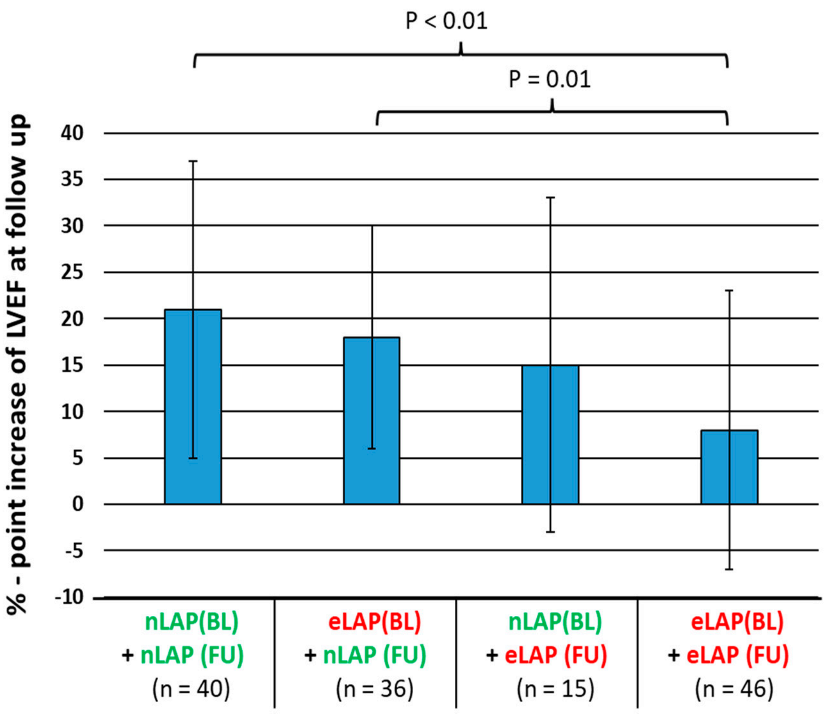

Supplementary figure S3. The association between estimated left atrial pressure (LAP) both at baseline (BL) and follow up (FU), and CRT induced LV reverse remodeling as expressed by the percentage point increase of left ventricular ejection fraction (LVESV) at follow up. eLAP: elevated LAP; nLAP: normal LAP.

Supplementary table S1. Univariable and multivariable linear regression analysis with relative decrease in left ventricular end-systolic volume (LVESV) as the dependent variable

	Univariable		Multivariable	
	Standardized coefficient	P-Value	Standardized coefficient	P-Value
Age at implantation	0.01	0.91	-0.10	0.21
Male sex	-0.19	<0.01	-0.18	0.05
ICM	-0.21	<0.01	-0.11	0.21
QRS width	0.16	0.04	0.07	0.36
NYHA	-0.12	0.09	-0.18	**0.02**
AF	-0.20	<0.01	-0.14	0.08
DM	-0.02	0.76	0.05	0.51
Serum Creatinine	-0.11	0.15	0.09	0.31
ACEi	-0.02	0.77	-0.13	0.13
BB	0.05	0.46	-0.04	0.67
LVEF	-0.20	<0.01	-0.14	0.10
nLAP (vs. iLAP and eLAP)	0.26	<0.001	0.20	**0.02**
LBBB	0.28	<0.001	0.22	**>0.01**

ICM: ischemic cardiomyopathy; NYHA: New York heart association; AF: atrial fibrillation; DM: diabetes mellitus; ACEi: angiotensin converting enzyme inhibitors; BB: B-blockers; LVEF: left ventricular ejection

fraction; LAP: left atrial pressure according to the guideline + LA strain algorithm; normal (n), indeterminant (i) and elevated (e); LBBB: left bundle branch block.

Supplementary table S2. Univariable and multivariable cox regression analysis for identifying predictors of survival (endpoint: all-cause mortality).

	Univariable		Multivariable	
	Unadjusted HR (95% CI)	P-Value	Adjusted HR (95% CI)	P-Value
Age at implantation	1.08 (1.04 – 1.11)	<0.001	1.08 (1.02 – 1.14)	**<0.01**
Male sex	1.74 (0.98 – 3.10)	0.06	0.81 (0.32 – 2.04)	0.65
ICM	1.86 (1.13 – 3.06)	0.02	0.99 (0.42 – 2.33)	0.97
QRS width	1.01 (0.99 – 1.03)	0.12	1.01 (0.99 – 1.03)	0.42
NYHA	1.63 (1.02 – 2.62)	0.04	1.21 (0.52 – 2.83)	0.66
AF	2.46 (1.33 – 4.54)	<0.01	4.10 (1.45 – 11.55)	**<0.01**
DM	1.31 (0.76 – 2.27)	0.33	1.09 (0.48 – 2.49)	0.82
Serum Creatinine	2.17 (1.58 – 2.98)	<0.001	1.83 (0.93 – 3.60)	0.08
ACEi	0.50 (0.27 – 0.95)	0.04	0.25 (0.53 – 3.03)	0.93
BB	0.51 (0.25 – 1.05)	0.07	0.66 (0.24 – 1.83)	0.43
LVEF	0.98 (0.97 – 1.12)	0.36	0.98 (0.95 – 1.02)	0.28
nLAP (vs. iLAP and eLAP)	0.40 (0.22 – 0.71)	<0.01	0.66 (0.29 – 1.45)	0.30
LBBB	0.42 (0.24 – 0.74)	<0.01	0.45 (0.21 – 0.95)	**0.04**

Hazard ratios (HR) are presented with 95% confidence interval (CI) in parentheses. ICM: ischemic cardiomyopathy; NYHA: New York heart association; AF: atrial fibrillation; DM: diabetes mellitus; ACEi: angiotensin converting enzyme inhibitors; BB: B-blockers; LVEF: left ventricular ejection fraction LAP: left atrial pressure according to the guideline + LA strain algorithm; normal (n), indeterminant (i) and elevated (e); LBBB: left bundle branch block.
